# Merkel Cell Polyomavirus in Cutaneous Swabs

**DOI:** 10.3201/eid1604.091278

**Published:** 2010-04

**Authors:** Vincent Foulongne, Nicolas Kluger, Olivier Dereure, Grégoire Mercier, Jean-Pierre Molès, Bernard Guillot, Michel Segondy

**Affiliations:** University of Montpellier I, Montpellier, France

**Keywords:** Merkel cell carcinoma, Merkel cell polyomavirus, MCPyV, tumors, skin diseases, viruses, dispatch

## Abstract

To assess the usefulness of using cutaneous swabs to detect Merkel cell polyomavirus (MCPyV) DNA, we analyzed swabs from persons with Merkel cell carcinoma (MCC), others with skin diseases, and healthy volunteers. MCPyV was detected in at least 1 sample from virtually all participants. Viral loads were higher in samples from patients with MCC.

Merkel cell polyomavirus (MCPyV) is a recently identified human virus initially discovered in tumor tissues of patients with Merkel cell carcinoma (MCC), a rare but aggressive skin cancer (*1*). Several studies have confirmed that MCPyV DNA is present in 70%–80% of MCC tumors (*1**–**4*) and that tumoral cells in most patients show a monoclonal integration of the viral genome and expression of the large T antigen (*1**,**5**–**7*). Furthermore, the integrated viral genome may harbor mutations in the T-antigen coding sequence, resulting in truncation of the corresponding helicase protein (*8*). These data support a causal role of MCPyV in tumorogenesis. However, the tumorogenic potential of MCPyV remains unclear because the MCPyV genome cannot be detected in 20%–30% of MCC tumors, whereas MCPyV DNA has been consistently identified in persons with other disorders or in the skin of healthy persons (*9**–**12*).

Most studies of MCPyV DNA detection in skin samples have been performed on biopsy samples. We assessed the relevance of using cutaneous swabbing instead of full-skin tissue samples for viral MCPyV DNA detection. Overall, our results show that MCPyV DNA was detected in cutaneous swabs from almost all study participants, which indicated that MCPyV is likely a ubiquitous virus.

## The Study

Cutaneous swabs were obtained from 46 persons: 5 patients with MCC (median age 76 years, range 68–78 years); 16 patients with various skin diseases (median age 76.5 years, range 41–90 years); and 25 clinically healthy volunteers (median age 35 years, range 22–58 years) who were recruited from among Montpellier University Hospital staff (Table). All study participants gave written informed consent, and the study was approved by the local ethics committee. Cutaneous swabs were collected from the face and, for most study participants, from the trunk (chest, back, or abdomen) and upper and lower limbs. Lesions in patients with MCC or other cutaneous disorders were also swabbed. Additionally, buccal mucosa swabs were obtained from 42 patients.

**Table:** Merkel cell polyomavirus DNA detection rates in swabs

Swab type	Patients with MCC* (no. positive/no. tested)	Patients with other skin diseases† (no. positive/no. tested)	Clinically healthy volunteers (no. positive/no. tested)
Buccal mucosa	1/5	0/13	1/24
Cutaneous			
Overall	27/29	58/78	56/70
Face	6/6	14/16	23/25
Trunk	11/11	16/20	13/16
Upper limb	1//2	10/15	9/14
Lower limb	7/8	10/15	11/15
Lesion	2/2	8/12	Not applicable

*MCC, Merkel cell carcinoma. †Patients with Kaposi sarcoma (n = 6), cutaneous drug side-effect (n = 3), leg ulcer (n = 3), bullous pemphigoid (n = 2), psoriasis (n = 1), and mycosis fungoides (n = 1).

Swabs were suspended in 400 μL of phosphate-buffered saline, and DNA was extracted from 300 μL of the suspension with an automatic EasyMag apparatus (bioMérieux, Marcy l’Etoile, France). The elution volume was 50 μL, and 10 μL of eluate was used for subsequent PCRs. An unused swab, processed in the same way as the cutaneous swab, was included in each run as the negative control. MCPyV DNA was detected by PCR by using large T antigen (LT3) and viral capsid protein (VP1) primers (*1*); MCPyV DNA levels were measured by real-time PCR as described (*11*). Total DNA level in swabs was measured by using the LightCycler Control DNA Kit (Roche Diagnostics, Meylan, France), and results were expressed as MCPyV DNA copies/ng DNA.

We analyzed categorical data by using the χ^2^ test. Continuous variables were compared by **using** the **Kruskal-**Wallis test for multiple groups and the Fligner-Policello rank tests for pairwise comparisons. Bonferroni correction was applied when appropriate. A p value <0.05 was considered significant.

Overall, MCPyV DNA was detected in at least 1 swab from all but 2 study participants (1 patient with Kaposi sarcoma and another with a leg ulcer). MCPyV DNA was detected in 141 (79.6%) of 177 cutaneous swabs and in 2 (4.8%) of 42 buccal mucosa swabs (p<0.001) (Table). The viral genome was detected 27 (93.1%) of 29 cutaneous swabs from patients with MCC, in 58 (74.3%) of 78 from patients with other cutaneous diseases, and in 56 (80.0%) of 70 from clinically healthy volunteers (p = 0.10). MCPyV DNA was more frequently detected on swabs taken from the face (43/47, 91.5%) than from the trunk (40/47, 85.1%) or limbs (48/69, 69.6%), but the difference was significant only between face and limb swabs (p = 0.005). The MCPyV genome was absent from swabs taken from 3 leg ulcers.

Levels of total human DNA were significantly higher in buccal mucosa swabs than in cutaneous swabs (2,170 pg/µL vs. 62 pg/µL; p<0.001). MCPyV DNA levels were higher in cutaneous swabs obtained from patients with MCC (median 861 copies/ng DNA) than in those from patients with other skin diseases (median 45 copies/ng DNA; p<0.001) or from the clinically healthy volunteers (median 43 copies/ng DNA; p<0.001) (Figure). MCPyV DNA levels were 5 and 11 copies/ng of DNA in each of the 2 positive buccal swabs.

**Figure:**
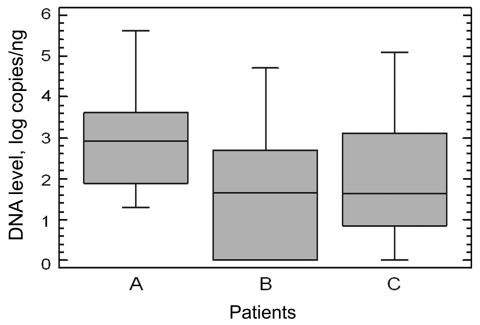
Comparison of Merkel cell polyomavirus DNA levels in cutaneous swabs obtained from 46 study participants. A) Patients with Merkel cell carcinoma; B) patients with other skin diseases; C) clinically healthy volunteers. Data are presented as box and whiskers plots. Boxes represent the interquartile range, lines within the boxes represent the median value, and whiskers represent 1.5 × the interquartile range.

## Conclusions

Our results demonstrate that MCPyV DNA can be efficiently detected by cutaneous swabbing. This easy method could be a useful tool for future epidemiologic or molecular studies targeting MCPyV. Indeed, this noninvasive procedure may be easily performed without the potential risk for side effects related to biopsy collection and is more acceptable than a biopsy for patients who do not have cutaneous disease. The high prevalence of MCPyV DNA at the skin surface, contrasted with its low prevalence in buccal mucosa and its absence in skin ulcers (where the epidermis is absent), strongly suggests that MCPyV is localized in the epidermis. As an alternative hypothesis, MCPyV could be released onto the skin surface through sebaceous or sudoral secretions. The relative high levels of viral DNA contrasted with the low amount of total DNA in cutaneous swabs might indeed support this hypothesis.

A lower prevalence of MCPyV DNA in skin biopsy samples among a similar subset of patients has been reported (*11*). However, this finding may be explained by the more limited amounts of superficial skin layers in skin biopsy samples compared with those in cutaneous swabs. Because disinfection of the skin before a biopsy may eliminate potential viral DNA from the epidermis, cutaneous swabbing may produce a more thorough sample for testing.

Our results are in accordance with those from a study by Loyo et al. that showed widespread distribution of MCPyV (*12*) and with those from a study by Wieland et al., who detected MCPyV DNA on the forehead skin of 62% of healthy persons (*13*). These findings strongly suggest that MCPyV is likely present in the skin of almost all adults. Furthermore, another recent study on MCPyV seroprevalence concluded that MCPyV circulates widely in the human population because specific MCPyV antibodies can be detected in as much as 80% of persons tested (*14*). Our results indeed support these serologic data and would be indicative of a persistent, although asymptomatic, infection with MCPyV in the epidermis of most persons.

Our results concur with those of other studies that found higher levels of MCPyV DNA in samples from MCC patients than from other subsets of patients (*11**,**12*). Similarly, higher MCPyV-specific antibody titers in patients with MCC have been reported (*14*). These consistent data suggest that MCPyV is likely involved in MCC pathomechanisms. However, high MCPyV DNA levels and high antibody titers (*11**,**12**,**14*) may be found in persons without MCC. The clinical relevance of increased viral load or antibody titers, therefore, remains unclear.

Our detection of MCPyV DNA in 2 buccal mucosa swabs is in line with recent reports of the widespread viral DNA in the human body (*12**,**13**,**15*). Even though these data might point toward a mucosal, respiratory, or fecal–oral route of transmission, the skin carriage we observed suggests that a cutaneous route should also be considered. Cutaneous swabbing could be an effective, less invasive method of detecting MCPyV DNA, providing a useful tool for future epidemiologic and molecular studies.
